# Influence of Bovine Whey Protein Concentrate and Hydrolysate Preparation Methods on Motility in the Isolated Rat Distal Colon

**DOI:** 10.3390/nu8120809

**Published:** 2016-12-14

**Authors:** Julie E. Dalziel, Rachel C. Anderson, Shalome A. Bassett, Catherine M. Lloyd-West, Neill W. Haggarty, Nicole C. Roy

**Affiliations:** 1 Food Nutrition & Health Team, Food & Bio-based Products Group, AgResearch, Grasslands Research Centre, Palmerston North 4442, New Zealand; rachel.anderson@agresearch.co.nz (R.C.A.); shalome.bassett@agresearch.co.nz (S.A.B.); nicole.roy@agresearch.co.nz (N.C.R.); 2 Bioinformatics and Statistics, AgResearch, Grasslands Research Centre, Palmerston North 4442, New Zealand; catherine.lloyd-west@agresearch.co.nz; 3 Fonterra Co-operative Group, Palmerston North 4442, New Zealand; neill.haggarty@fonterra.com; 4 Riddet Institute, Massey University, Palmerston North 4442, New Zealand

**Keywords:** milk, cheese, peptide, contraction, intestinal transit, trans-epithelial electrical resistance, immune modulation

## Abstract

Whey protein concentrate (WPC) and hydrolysate (WPH) are protein ingredients used in sports, medical and pediatric formulations. Concentration and hydrolysis methods vary for whey sourced from cheese and casein co-products. The purpose of this research was to investigate the influence of whey processing methods on in vitro gastrointestinal (GI) health indicators for colonic motility, epithelial barrier integrity and immune modulation. WPCs from casein or cheese processing and WPH (11% or 19% degree of hydrolysis, DH) were compared for their effects on motility in a 1 cm section of isolated rat distal colon in an oxygenated tissue bath. Results showed that WPC decreased motility irrespective of whether it was a by-product of lactic acid or mineral acid casein production, or from cheese production. This indicated that regardless of the preparation methodology, the whey protein contained components that modulate aspects of motility within the distal colon. WPH (11% DH) increased contractile frequency by 27% in a delayed manner and WPH (19% DH) had an immediate effect on contractile properties, increasing tension by 65% and frequency by 131%. Increased motility was associated with increased hydrolysis that may be attributed to the abundance of bioactive peptides. Increased frequency of contractions by WPH (19% DH) was inhibited (by 44%) by naloxone, implicating a potential involvement of opioid receptors in modulation of motility. Trans-epithelial electrical resistance and cytokine expression assays revealed that the WPC proteins studied did not alter intestinal barrier integrity or elicit any discernible immune response.

## 1. Introduction

Whey protein has historically been produced in the dairy industry as a by-product of casein or cheese production and is now recognized as a nutritious healthy protein source [1]. When processed into whey protein concentrate (WPC) it is used as a health or sports supplement. Whey protein hydrolysate (WPH) is used for pediatric formulas due to its nutritionally available amino acid composition and low allergenicity. Whey is generally considered a dietary protein supplement that can provide antimicrobial activity, immune modulation, improve muscle strength and body composition and protects against cardiovascular disease and osteoporosis [2,3,4,5]. Both WPC and WPH are used as a protein source in sports nutrition supplements where their rapid digestion is considered helpful for increasing skeletal muscle protein synthesis [6]. Whey is considered a fast protein in that it reaches the jejunum rapidly compared with casein, but then hydrolysis is slower, allowing greater absorption over the length of the small intestine [2]. However, the ability of whey to alter gastrointestinal (GI) transit is not well understood.

Whey comprises 0.7% (*w*/*v*) of milk content compared with 2.8% for casein. The major whey proteins in bovine milk are β-lactoglobulin (50%–55%), and α-lactalbumin (20%–25%). Other whey proteins include immunoglobulins (10%–15%), serum albumin (5%–10%), lactoferrin (1%–2%), enzymes, hormones, growth factors, nutrient transporters, and disease resistance factors [7,8]. Because most of the commercial WPC products are repurposed from other dairy processes, they will differ in their protein/peptide composition and physical properties such that not all WPCs can be considered identical. It is not known if they modulate GI function in a similar manner.

Separation of whey and casein components from skim milk involves specific processes that relate to the intended use of the end product and several different methods are therefore employed to precipitate the casein. For example, intact whey obtained as a by-product of casein production is prepared by a number of methods. One method uses addition of mineral acid (sulfuric or hydrochloric) to lower the pH to 4.6 to precipitate the casein. Another method uses lactic acid bacteria (LAB) cultures to convert lactose to lactic acid which lowers the pH to precipitate the casein. Whey is also produced as a by-product of cheese production, where the whey is separated from casein using LAB precipitation in conjunction with rennet. In this case, rennet (containing the protease chymosin), hydrolyses casein to release a large peptide from kappa-casein known as glycomacropeptide (GMP) which comprises 10%–15% of the cheese whey protein [7]. GMP release causes the casein to precipitate as the first step in cheese production. This is the basis of standard cheese production that results in a by-product known as cheese or sweet whey.

Some infant formulas use whey protein that has been further processed by enzymatic hydrolysis to produce more easily digestible protein, which can improve management of common GI symptoms and allergic disease [9]. Little is known about the effect of whey proteins, either intact or hydrolysed, on GI motility and transit. This is because few studies in healthy infants have been published that compare these whey proteins directly without changing other formula components [10,11]. Where this has been investigated, hydrolysed proteins accelerate GI transit of milk and stools in preterm infants [12].

Given their use in high performance sport nutritional supplements and pediatric formulas, the wider effects of WPC and WPH on GI health beyond protein and basic nutrition is of broad interest. This includes GI wall motility and resilience of the protective cell barrier layer. The aim of this research was to investigate the influence of whey protein production methods on GI health indicators of colonic motility and epithelial barrier integrity. We hypothesise that intact whey alters colonic motility and that hydrolysed whey will have a greater effect due to the higher levels of peptides present. Muscle contraction assays are in routine use in the pharmaceutical industry to identify active compounds involved in GI motility [13,14,15], but have not been widely used for food research. We used a rat model [16,17,18] because many dietary preclinical studies are done in this species and this would enable comparison with other studies. Bioassays to measure the effects of the WPC ingredients on trans-epithelial electrical resistance (TEER) across an epithelial cell layer and cytokine production by peripheral blood mononuclear cells (PBMCs) provided additional information on barrier function.

## 2. Materials and Methods

### 2.1. Animals

This study was carried out in strict accordance with the recommendations of the New Zealand Animal Welfare Act 1999. The tissue collection protocol was approved by the AgResearch Limited (Grasslands) Animal Ethics Committee (Ethics Approval Numbers: AE72 & AE98). Animals were euthanized by CO_2_ inhalation overdose. Male adult Sprague-Dawley rats, 3–9 months of age, weighing 250–550 g were obtained from the AgResearch Ruakura Small Animal Unit (Hamilton, New Zealand), housed under a 12 h light/dark cycle, and fed Sharpes Diet 86 (Sharpes Stockfeeds Ltd, Carterton, New Zealand).

### 2.2. Preparation and Composition of Whey Protein

Bovine milk-derived whey proteins were provided by the Fonterra Research and Development Centre (Palmerston North, New Zealand) prepared by the methods outlined below and supplied as dried powder (Table 1). All powders were reconstituted in water at 1 mg/mL for the purposes of these experiments.

#### 2.2.1. Whey Protein Concentrate

WPC was prepared by three different methods common to the dairy industry and concentrated to 80% total solids. WPC-L was produced from milk using LAB cultures to convert lactose to lactic acid to lower the pH to 4.6 in order to precipitate the casein. WPC-LR was produced using a combination of rennet and LAB. The rennet enzyme mixture had been inactivated during processing. WPC-MA was produced using mineral acid, sulfuric or hydrochloric, to lower the pH (~pH 4.6) in order to precipitate the casein.

#### 2.2.2. Whey Protein Hydrolysate

WPH was produced from WPC-L using two different enzymatic digestion methods that resulted in different extents of hydrolysis, and therefore a different spectrum of peptides (Table 2). The degree of hydrolysis was determined by the modified O-phthaldehyde (MOPA) method [19]. WPH-P was 11% hydrolysed using porcine pancreatin. WPH-AT was 19% hydrolysed using alcalase and thermoase. The molecular weight profile of the hydrolysates was determined using a Tosoh Gel Permeation TSK G2000 SWXL 30 cm column; elution used solvent of 0.1 M potassium phosphate, 0.3 M KCl, 15% acetonitrile, pH 7; flow rate of 0.5 mL/min; detection at 205 nm; isocratic elution over 40 min. Sample concentration of 1 mg protein/mL elution solvent, with a 50 μL injection.

### 2.3. Modulators

Naloxone hydrochloride dihydrate was purchased from Sigma-Aldrich (St. Louis, MO, USA) and stored as a 10 mM frozen stock −20 °C that was diluted in Krebs buffer immediately prior to use. Acetylcholine chloride was purchased from Sigma-Aldrich (St. Louis, MO, USA) and diluted from a 1 mM stock.

### 2.4. Distal Colon Motility

Methods were similar to those described previously for the short distal colon [16,17,18]. After euthanasia, a 4 cm piece of distal colon was removed 1 cm distal to the striations of the mid colon. The tissue was placed in Kreb’s buffer (118 mM NaCl, 4.7 mM KCl, 1.2 mM KH_2_PO_4_, 1.2 MgSO_4_ mM, 2.6 mM CaCl_2_, 25 mM NaHCO_3_, 11 mM glucose, pH 7.4) oxygenated with 95% O_2_/5% CO_2_. The preparation was divided into four pieces of equal length and each distal colon piece was mounted longitudinally on holders in an isolated tissue bath system (SI-MB4, SI-Heidelberg, World Precision Instruments, Sarasota, FL, USA), at 37 °C. Tissues were suspended under 2 g of tension and equilibrated for one hour in Krebs buffer during which time the bath solution was exchanged every 15 min. Spontaneous muscle contractions vary among muscle preparations in their tension and frequency. Thus, baseline recordings measured prior to any treatments were measured as controls during the last 10 min of a one hour exposure to Krebs buffer following equilibration and compared with the last 10 min of the response to 15 min exposure to the test treatments. Test treatments were added to the external tissue preparation by bath perfusion. This mimics in vivo exposure following absorption from the small intestine and reaching the colon via the circulatory system. Smooth muscle contraction data were measured using BAM21-E amplifiers integrated using Lab-Trax 4/24T hardware and acquired and analysed using Labscribe 2 software (iWorx Inc., Dover, NH, USA). To allow for recovery from the solution exchange, data from the last 10 min of a 45 min recording period were analysed and the mean for contractile amplitude of frequency was determined from the contractile responses recorded. Contractile amplitude (*g*) was measured from the baseline to the maximum peak of the contractile tension response. Contractile frequency was measured as the number of contractions counted per min. The maximum amplitude was compared to that in the pretreatment control, and preparations that gave little or no excitatory smooth muscle contractile response to 1 µM acetylcholine at the end of the experiment were excluded because this indicated that the tissue was no longer viable.

When naloxone was used, the test protein was first added alone followed by 45 min of wash out, then naloxone was added followed by 45 min of wash out, then both the protein and the naloxone were co-applied followed by 45 min of wash out.

### 2.5. Intestinal Barrier Integrity

To determine whether the compositional differences resulted in differences in effects on intestinal barrier integrity, the TEER across an in vitro GI epithelial layer was measured in response to selected WPC proteins. The ability of the WPC proteins to overcome the decrease in TEER caused by the pro-inflammatory cytokine tumor necrosis factor alpha (TNFa) was also investigated. A human colorectal adenocarcinoma cell line (Caco-2 cells, ATCC HTB-37) was used as a model of human GI epithelium because these cells differentiate into polarized epithelial cells possessing apical brush borders and intracellular tight junctions [20,21]. Caco-2 cells were seeded at a density of 8 × 10^4^ cells/Transwell (6.5 mm, polyester, 0.4 µm pore size; Corning Incorporated, Corning, NY, USA) and grown in Medium 199 (M199; Gibco, Invitrogen Corporation, Carlsbad, CA, USA) supplemented with 10% fetal bovine serum (FBS; Gibco, Invitrogen Corporation Carlsbad, CA, USA), 1% non-essential amino acids (NEAA; MEM non-essential amino acids 100× solution; Sigma-Aldrich) and 1% penicillin–streptomycin (Pen–Strep: 10,000 units/mL penicillin G sodium salt and 10,000 µg/mL streptomycin sulfate in 0.85% saline; Gibco, Invitrogen Corporation) at 37 °C in 5% CO_2_ for 15 to 19 days until they formed a differentiated monolayer. The day prior to the TEER assay, Transwells were transferred into cellZscope cell modules (nanoAnalytics GmbH, Münster, Germany) with M199 supplemented with 10% FBS, 1% NEAA and 1% Pen–Strep in the basal compartment and M199 supplemented with 1% NEAA in the apical compartment, and incubated (37 °C, 5% CO_2_). The background resistances across the cell layers were measured hourly for 24 h using the cellZscope computer-controlled system. The TEER values were calculated by the cellZscope software (v 2.2.0) by multiplying the raw resistance by the membrane area (0.33 cm^2^). Caco-2 monolayers that had background TEER greater than 400 ohms/cm^2^ were used for assays.

The apical medium from the Transwells was replaced with the treatment solutions (*n* = eight per treatment): control medium (M199 supplemented with 1% NEAA), control medium containing 1 mg/mL WPC-L, or control medium containing 1 mg/mL WPC-LR. The basal medium from the wells was replaced with M199 supplemented with 10% FBS, 1% NEAA and 1% Pen–Strep for half of the inserts (no challenge) and the same solution containing 100 ng/mL TNFa for the other half of the inserts (TEER-reducing challenge). TEER was measured every hour for 48 h and the percentage change in TEER at each time point compared to the initial TEER (before treatments were added) was calculated. Three independent assays, each with four replicates per treatment, were carried out (total *n* = 12 per treatment).

### 2.6. Cytokine Expression

To determine whether there was a difference in the immune stimulatory properties of the whey proteins [3], cytokine secretion by PBMCs was measured. Written consent for collection and use of blood for research purposes was obtained from human volunteers. Ethics approval from the New Zealand Health and Disability Committee was not required under Section 3 of the Standard Operating Procedures of the Health and Disability Committee due to the healthy status of the volunteers and the anonymity of the samples, and the fact that the PBMCs were used as a reagent in the experiment rather than the subject of the experiment.

The method used has been described previously [22]. In brief, PBMCs were isolated from the blood of five male adult donors using the Ficoll Paque method as per the manufacturer’s instructions (Sigma-Aldrich). PBMCs were seeded into 96-well plates at a density of 5 × 10^5^ cell/mL and cultured in Advanced Roswell Park Memorial Institute medium (RPMI) (Invitrogen Corporation) supplemented with 2% FBS and 1% Pen–Strep at 37 °C in 5% CO_2_. After 24 h, the medium was replaced with the treatment solutions of either control medium, or medium containing 2.5–12.5 µg/mL phytohemagglutinin (PHA, Sigma-Aldrich), or 0.01–0.5 ng/mL lipopolysaccharide (LPS, Sigma-Aldrich), or 0.1 mg/mL whey protein (eight replicates × five donors for each whey protein). PHA and LPS treatments were positive controls to show that the T and B lymphocytes present within the PBMC population were viable and responsive to stimuli. After 24 h incubation at 37 °C in 5% CO_2_, the cell culture supernatants were collected.

The concentrations of seven cytokines (IL-4, IL-8, IL-10, IL-12p70, IL-17F, TNFa, IFN-γ) were determined using a Cytometric Bead Array (CBA) Flex Set (BD Bioscience; Becton-Dickinson, San Jose, CA, USA) as per the manufacturer’s instructions. Analysis of the resulting fluorescence-activated cell sorting data to determine individual cytokine levels was performed using FCAP Array V3.0™ (BD Bioscience). Results for each donor were normalized against the medium controls and data were expressed as a fold change in secretion compared to the control medium.

### 2.7. Statistical Analysis

#### 2.7.1. Colonic Motility

The response variables Tension and Frequency were normalized within each animal to take into account the variability between animals. Ratios for each time point after the treatment had been applied were calculated by dividing the tension or frequency by the value at the initial time point. The ratio was log-transformed in order to meet the requirements of ANOVA. Data were analysed using a Repeated Measures Linear Mixed Model (via REML) to examine the effects of the factors Treatment (WPC-MA & WPH-AT) and Time (15, 30, 45, 60, 120 min). The data in Table 3 were analysed using a one-way ANOVA for the factor Treatment (7 levels). The Bonferroni-adjusted confidence intervals of the ratios at each time point for each Treatment were examined. If the confidence intervals included zero, then the response was significantly different to the value at Time 0. The analyses were carried out using GenStat version v18 (VSN International Limited, Hemel Hempstead, UK).

#### 2.7.2. Intestinal Barrier Integrity

The changes in TEER caused by the different treatments were compared in GenStat v17.1 (VSN International Limited, Hemel Hempstead, UK) using a Linear Mixed Model (via REML) analysis with an auto-regression order 1 (AR-1) covariance model to take account of the repeated measures. If the treatment × time interaction effect was significant (*p*-value < 0.05), the means of individual treatments at a given time point were compared using Fisher’s least significant difference (LSD) at 5%.

#### 2.7.3. Cytokine Expression

Treatments were compared using one-way ANOVA, where a *p*-value less than 0.05 was considered significant, followed by a Bonferroni post-hoc test.

## 3. Results

### 3.1. Whey Protein Concentrate

**Figure 1:**
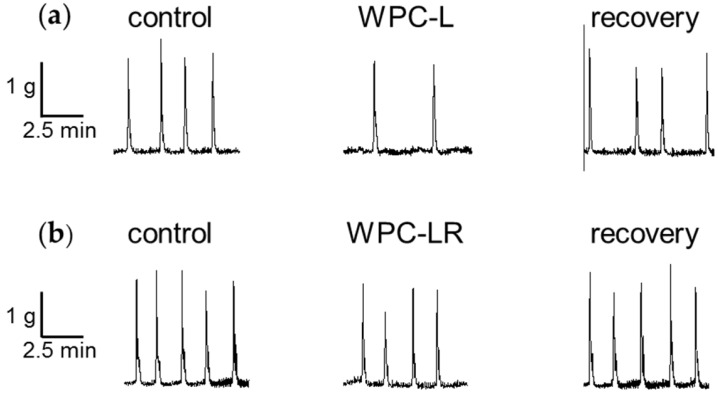

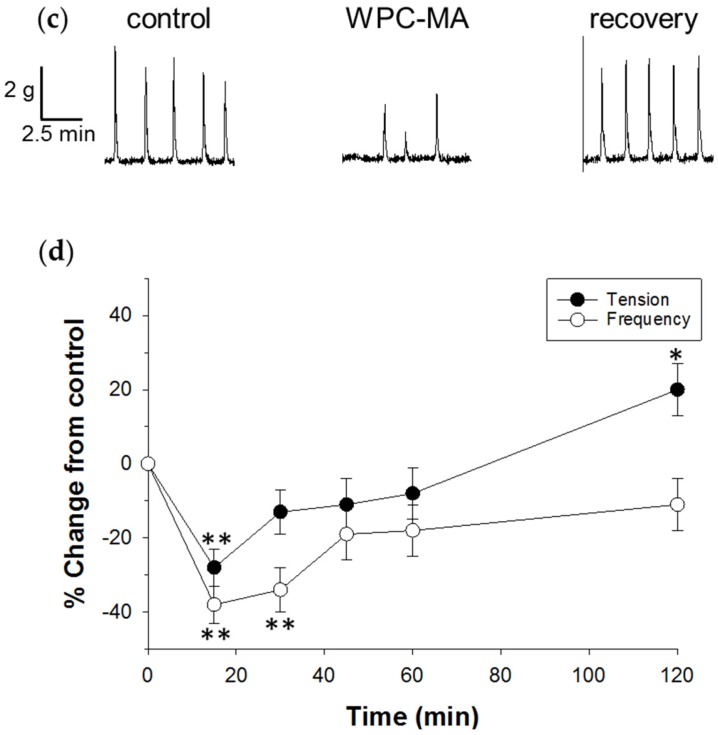
Effect of WPC on spontaneous muscle contractions in the rat distal colon. The last 10 min of a 15 min treatment exposure on spontaneous muscle contractions was measured relative to that in the last 10 min of a 60 min pretreatment control recording (*T* = 0). Examples of raw data recordings from three separate tissue preparations show changes in muscle tension over time for the control, after addition of 1 mg/mL: (**a**) WPC-L; *n* = 13; (**b**) WPC-LR; *n* = 15; and (**c**) WPC-MA; *n* = 12, and the last 10 min of recovery following a 60 min washout in Krebs buffer; (**d**) Time-dependence of WPC-MA effect shown as percent change in tension and frequency relative to the pretreatment control for a 60 min application showing pooled data from 12 experiments. Data show mean ± SEM. Asterisks indicate statistical significance (* *p* < 0.05, ** *p* < 0.01) of the treatment relative to control.

Spontaneous contractions were recorded from the isolated rat distal colon in vitro before, during and after addition of the WPC test treatment (Figure 1). Contractile parameters were measured before and after addition of the test treatment applied to separate preparations for 15 min, followed by 45 min in Krebs buffer. The last 10 min of a 15 min treatment exposure on spontaneous muscle contractions was measured relative to that in the last 10 min of a 60 min pretreatment control recording. The three WPCs were compared to determine how their different methods of preparation (Table 1) might affect contractility in the isolated distal colon.

A summary of the results showed that WPC-L and WPC-LR decreased the frequency of contractions and WPC-MA decreased the frequency and tension of contractions (Table 3). Smooth muscle contractions were not different from control levels after 45 min wash out in Krebs buffer for either WPC. Because WPC-MA had the greatest inhibitory effect, the time-course of inhibition was examined over one hour and the contractile response was monitored (Figure 1d). The decrease in tension and frequency was maximal after 15 min of exposure to WPC-MA and was short-lived. Frequency returned to levels similar to the pretreatment control following wash out with Krebs buffer, but tension increased.

**Figure 2:**
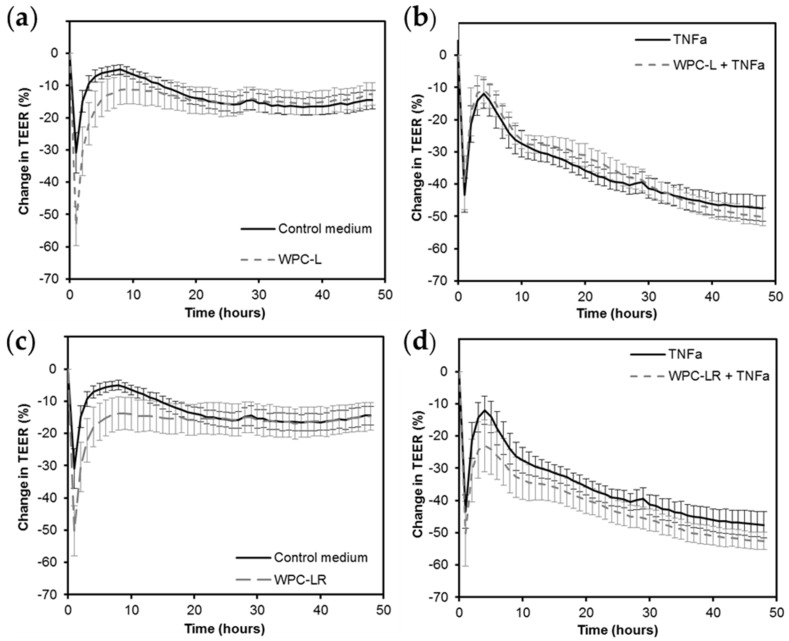
Effect of WPC on the trans-epithelial electrical resistance (TEER) across Caco-2 cell monolayers, (**a**,**c**) under normal conditions, and (**b**,**d**) challenged with tumor necrosis factor alpha (TNFα) for WPC-L and WPC-LR. The values shown at the % change in TEER compared to the initial TEER for each insert. The data from three independent assays, each with four replicates per treatment, were combined (total *n* = 12 per treatment) to calculate the mean ± SEM. There were no significant differences between treatment groups and their respective controls.

Because WPC-L and WPC-LR were produced using biologically active LAB and are expected to contain non-replicating microbes, we investigated whether they affect TEER across Caco-2 cell layers and the release of cytokines by human PBMCs, respectively. The controls behaved as expected. The unchallenged control had an initial drop in TEER due to the change in solution and then recovered to the initial TEER. The TNFa challenged control had reduced TEER over time. Neither WPC-L nor WPC-LR altered TEER compared to control medium or overcame the drop in TEER caused by TNFa (Figure 2). Immune effects were not studied for WPC-MA as this whey protein was chemically rather than biologically prepared using cultures or enzymes. Whilst IL-8 levels increased in WPC-L and WPC-LR treated cells (>2 fold) as measured by flow cytometry, the other cytokines remained below detectable levels (data not shown). This shows that overall the whey proteins tested did not drive cytokine expression changes in PBMCs.

### 3.2. Whey Protein Hydrolysate

**Figure 3:**
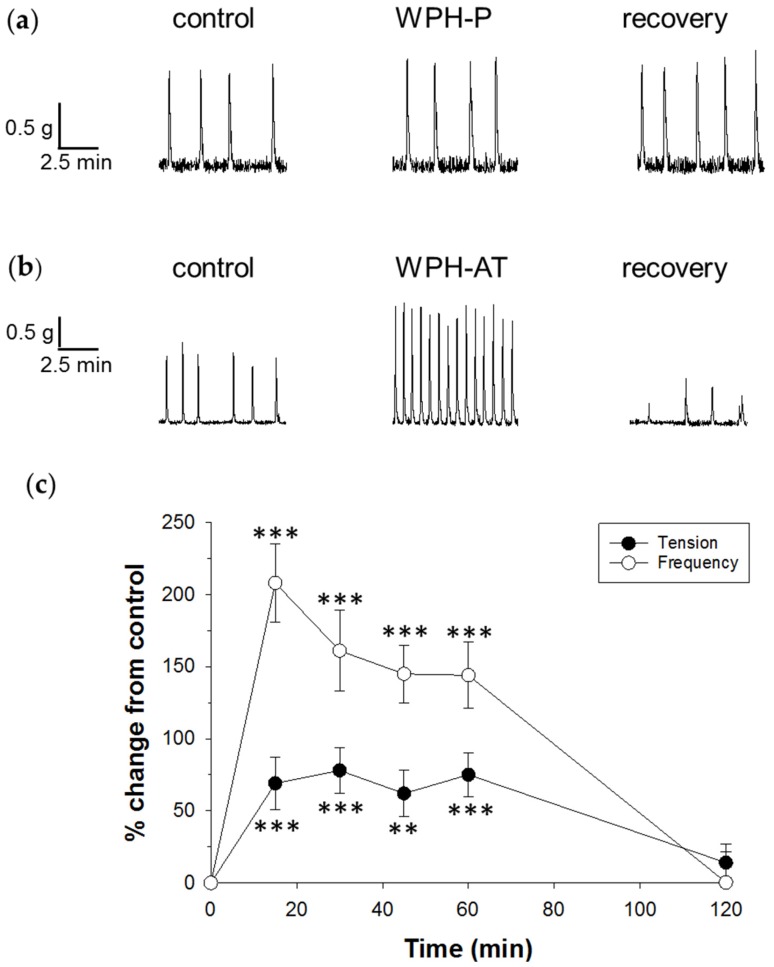
Effect of WPH on spontaneous smooth muscle contractions in the rat distal colon. Examples of raw data recordings from two separate experiments show changes in muscle tension over time for the control, after addition of 1 mg/mL (**a**) WPH-P; *n* = 12; (**b**) WPH-AT; *n* = 11, and the last 10 min of recovery following a 60 min washout in Krebs buffer; (**c**) Time-dependence of WPH-AT effect shown as percent change in tension and frequency from the pretreatment control for a 60 min application showing, pooled data from 12 experiments. Treatment with WPC-AT was applied for 60 min (*n* = 11). Data show mean ± SEM. Asterisks indicate the significance of each treatment relative to controls (** *p* < 0.01, *** *p* < 0.001).

Because WPC-L produced a significant decrease in colonic rhythmicity, it was chosen for further study in its hydrolysed forms. To investigate whether different methods of hydrolysis of WPC would alter motility, two hydrolysed whey products made using different proteolytic enzyme systems were compared. The two enzyme systems used resulted in WPH with different degrees of hydrolysis; WPH-P that was 11% hydrolysed and WPH-AT that was 19.4% hydrolysed. Also, the two enzyme systems used will have different specificities, hydrolyzing the protein at different peptide bonds. This will result in these two hydrolysates having different peptide profiles (Table 2) that may affect motility. WPH-P did not alter motility during exposure in the tissue bath, but following washout the tension increased by 27% ± 11% (*n* = 12) (Figure 3a).

In contrast, WPH-AT produced a 65% increase in tension and a 131% increase in frequency (Figure 3b, Table 3). To investigate the time-course of this effect, WPH-AT was applied for 60 min (Figure 3c). This showed that the maximal effect on tension occurred between 5 and 15 min of treatment exposure and was sustained during the remaining treatment period, then returned to baseline level following washout. Similar results were found for frequency, with the exception that the maximal effect appears to have occurred between 20 and 30 min of treatment exposure. To rule out artefacts of hydrolysis processing on motility, the enzymes (active and inactive forms) used to produce WPH-AT were examined and found not to alter colonic motility when applied separately (Table 3). (WPH-P hydrolysis enzymes were not tested because this whey protein had already undergone extensive biological testing.)

**Figure 4:**
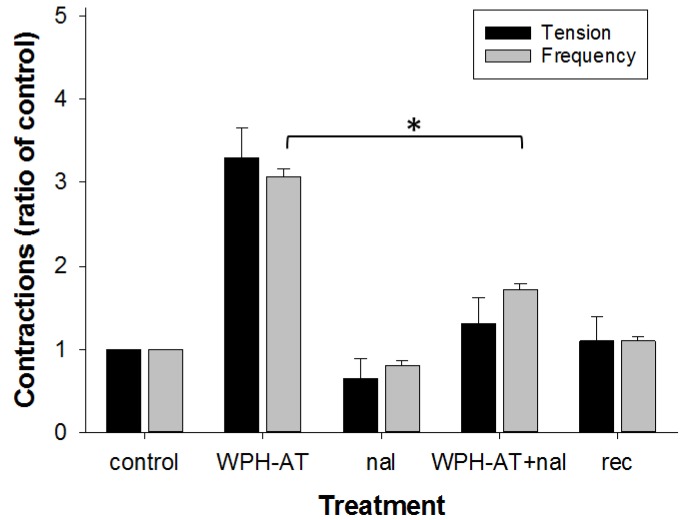
Effect of an opioid antagonist on the WPH-induced response in the rat distal colon. The effect of 1 µM naloxone (nal) on the WPH-AT-induced increase in contractile tension and frequency is shown as a ratio of the pretreatment control where treatments were applied consecutively to each preparation, averaged over the preceding 10 min of a 15 min application. (*n* = 3). Data show mean ± SEM. Asterisk indicates significance difference (* *p* < 0.05).

To determine whether the increased contractile frequency effect of WPH-AT involved an opioid agonist effect, the opioid antagonist naloxone was applied (Figure 4). The response to WPH-AT plus naloxone was reduced by 44% compared to that for WPH-AT alone, demonstrating that pre-exposure to 1 µM naloxone partially inhibited the WPH-AT-induced increase in spontaneous contraction frequency in the distal colon. The response to naloxone (1 µM) applied alone was similar to that for control Krebs buffer as expected. Because WPH-AT was derived from WPC-L, the ability of naloxone to prevent the reduced frequency response induced by the intact parent substrate was examined. The contractile response to WPC-L plus naloxone was not different from that for WPC-L alone (*n* = 3), suggesting that WPC-L lacked agonist opioid activity.

## 4. Discussion

The results of this study demonstrate that the ability of whey protein to alter GI motility is independent of the method of production, but does vary with the method of hydrolysis. The finding that the production method for whey protein was not a qualitative determinant of their biological action on colonic motility has not been reported previously. Our data indicate that WPC altered colonic motility, when it was either a co-product of acid casein production (WPC-L and WPC-MA) or from cheese production (WPC-LR). These results support our hypothesis in demonstrating that WPC can have modulatory effects on colonic motility, and indicates that this is independent of the method of precipitation used to remove the casein. In the relatively short segments of distal colon studied, the effects of WPC on motility were inhibitory. In contrast, WPH (WPH-AT) was found to act as a prokinetic and increased motility. This prokinetic action by WPH-AT may correlate with the release of specific bioactive peptides as a result of hydrolysis from alcalase and thermoase as opposed to porcine pancreatin. The lack of change in TEER across Caco-2 cell layers or cytokine production in PBMCs, indicated a healthy epithelial cell barrier layer that was not affected by WPC-L and WPC-LR and supports the food safe rating of these LAB starter culture produced whey proteins. In a normal inflammatory situation, a spectrum of inflammatory cytokines are upregulated. However, in our study, only IL-8 was increased, therefore the change in IL-8 is unlikely to be physiologically relevant. Although, overall, we did not detect a significant response in cytokine production, a recent report has shown that under controlled conditions, particularly for temperature, it is possible to preserve the biological activity of heat-sensitive factors in whey [23]. However, the results we detected for the commercial whey by-products that were used in our study are comparable with the results obtained for the heat treated commercial products tested in the study of Nguyen et al. (2016) [23] where increased IL-8 in response to WPC ingredients was observed.

### 4.1. Whey Protein Concentrate

The main effect of WPC was an inhibitory effect on motility of the isolated colon preparation. An active effect of WPC on motility might arise through low level release of peptides during processing. However, use of LAB may result in a small degree of whey protein hydrolysis that may result in the free peptide composition of lactic acid whey being different to that of mineral acid whey. Such a possibility does not appear to have conferred enhanced motility actions to WPC-L and WPC-LR over that of WPC-MA, as one may have anticipated. The reason why the inhibitory effect of WPC-LR was substantially less than that for WPC-L and WPC-MA remains unclear. The simplest explanation is that the presence of the rennet enzyme in the reaction to produce WPC-LR contributed to reducing the decrease in motility that occurred for WPC-L and WPC-MA. The presence of the rennet enzymes (containing the protease chymosin) used in WPC-LR production were inactivated during processing. Hence, they are not expected to be responsible per se for a reduced effect of WPC-LR on motility. The WPC-LR production method, however, utilizes whey as a co-product of cheese-making; therefore the rennet used to hydrolyze casein from skim milk releases a large peptide from kappa-casein called glycomacropeptide (GMP), which causes the casein to precipitate [7]. The whey resulting from the WPC-LR process is therefore expected to differ from WPC-L and WPC-MA in protein composition due to the presence of GMP that is co-concentrated with the remaining whey proteins. GMP might contribute to the reduced inhibitory effect of WPC-LR on colonic motility by diluting out an active component (4/5 dilution), relative to WPC-L and WPC-MA that do not contain GMP. Alternatively, GMP might counter an inhibitory effect of other components in WPC-LR.

It is also possible that for WPC-LR, proteolysis by the rennet enzymes resulted in the release of peptides from whey proteins including β-lactoglobulin. For instance, the release of the opioid agonist peptide β-lactorphin (among others) from whey protein stimulates contractions of guinea pig ileum in vitro [24]. Such a promotility effect of this peptide in WPC-LR might partially counter the inhibitory effects from other peptides, explaining its reduced effect compared with the other two casein co-product WPCs.

The results suggest that the LAB reaction and the MA reaction produced a common change in the whey protein, such as release of low levels of peptides, which decreased the frequency of contractions. The additional effect of WPH-MA of decreased contractile amplitude, which did not occur for WPH-L or WPH-LR suggests an additional inhibitory effect.

### 4.2. Whey Protein Hydrolysate

The results for the whey hydrolysates indicate that hydrolysis of WPH generates two different biologically active peptide profiles (using two different proteolytic enzyme systems) that result in different effects on up-regulation of motility. The gradual increase in tension by WPH-P might reflect a lower concentration of peptides and therefore a lower dose of active modulators to the distal colon preparation. The lack of an immediate prokinetic response to WPC-L and WPH-P, combined with no effect of AT, suggests that the prokinetic effects are likely to be a result in the difference in protein profiles which occur from AT digestion of WPC-L protein.

The opposite direction of the motility changes for WPH compared with WPC suggests that different peptides with different biological activity may be present. The peptides resulting from hydrolysis are therefore having a different net effect on colonic motility compared with that produced from low level WPC hydrolysis. Obviously, because WPC is hydrolysed, WPH contains many more peptides.

WPC-AT is not a pure fraction but a mixture containing many peptides, and the usefulness of antagonists to tease out the mechanism may therefore be limited. However, the prokinetic effects of WPC-AT are similar to our previous report for an opioid agonist peptide in the distal colon [18] suggesting that the partial inhibitory effect of naloxone might implicate an opioid agonist effect of WPC-AT. The lack of effect of naloxone on the response to the intact parent substrate WPC-L suggests that the effect is likely to be induced by a peptide hydrolysis product.

We note that in a full length large intestine preparation, an increase in frequency by an opioid agonist peptide was localized to the distal colon and that the overall effect of the opioid agonist peptide was to inhibit propagating contractions [18]. Thus, increased frequency of contractions in this short segment of the colon might not necessarily translate to faster GI transit. Our results do however demonstrate bioactivity and suggest that further investigation of whey protein hydrolysate mechanisms of action is warranted.

We have demonstrated previously that spontaneous phasic contractions in short segments of distal colon are independent of enteric neural activity, as they persist in the presence of tetrodotoxin (TTX) [16]. These contractions are therefore of myogenic rather than neural origin. However, we also showed that TTX increases the tension and frequency of contractions [16] suggesting that inhibitory connections predominate in the enteric neuronal network of this distal colon segment. Thus, it is possible that the WPH was able to modulate either muscle or remaining inhibitory neural activity. This means that a limitation of the technique used in this work is that the direction of modulation (increased or decreased motility) does not directly relate to the ability to increase or decrease total transit time, but may be a contributing factor in the distal colon region only.

### 4.3. In Vivo Implications

Milk proteins are digested in the stomach and small intestine to release physiologically active peptides. Our research suggests that some peptides may be present in the whey proteins studied prior to stomach digestion, thus if they are absorbed and reach the peripheral circulation would be active at modulating colonic motility. We note that WPC-LR has already been exposed to the stomach enzyme chymosin during processing, and thus may be considered partially pre-digested. It is possible that further release of other peptides by other GI enzymes might alter the overall modulatory outcomes in vivo. It will be necessary to examine how ingestion of these whey proteins alters GI transit in vivo to corroborate the modulatory outcomes that our in vitro data predict. Nevertheless, the slowing effect of the WPC casein co-products on distal colon contractile frequency compared with increased frequency for the corresponding WPH, would be consistent with accelerated GI transit in preterm infants fed formula containing hydrolysed compared with non-hydrolysed whey [12]. However, in infants, it has been shown that small amounts of intact protein may survive digestion [25] and could reach the colon and affect motility.

## 5. Conclusions

Our study demonstrates that whey co-product processing methods used for the concentration and hydrolysis of dairy ingredients are important determinants of the modulatory actions of whey on GI motility in a rat distal colon in vitro model. Thus, the whey source may become a consideration for future end-product use in functional foods.

## Figures and Tables

**Table 1:** Whey protein composition (average).

Nutrients (*w*/*w*%)	WPC-L	WPC-LR	WPC-MA	WPH-P ^1^	WPH-AT ^1^
Total protein	80	80	80	80	77
**% protein**					
α-lactoglubulin	16	13	16	ND	ND
β-lactoglubulin	58	43	52	ND	ND
BSA	2	2	2	ND	ND
IgG	4	4	6	ND	ND
GMP	0	21	0	ND	ND
Other	20	17	24	ND	ND
Lactose	4–6	4–6	4–6	1	4
Fat	4	5	5	4	6
Ash	3–9	3–9	3–9	4	9
Calcium (mg/100 g)	240	400	230	43	1
Moisture	3	4	4	4	ND

^1^ Derived from WPC-L. WPC-L, whey protein concentrate—lactic acid; WPC-LR whey protein concentrate—lactic acid and rennet; WPC-MA, whey protein concentrate—mineral acid; WPH-P, whey protein hydrolysate—pancreatin; WPH-AT, whey protein hydrolysate—alcalase and thermoase; BSA, bovine serum albumin; GMP, glycomacropeptide (determined by HPLC); ND, not determined.

**Table 2:** Hydrolysate molecular weight profile (average).

Mass Range (Daltons)	WPH-P % Protein	WPH-AT % Protein
>20,000	44	13
5000–20,000	17	20
1500–5000	16	23
1000–1500	6	8
500–1000	8	12
<500	9	23

WPH-P, whey protein hydrolysate—pancreatin (degree of hydrolysis, 11%); WPH-AT, whey protein hydrolysate—alcalase and thermoase (degree of hydrolysis, 19%).

**Table 3:** Effect of whey proteins on distal colon motility.

Whey	Tension	Frequency	*n*
WPC-L	↑ 5 ± 6	↓ 48 ± 12 ***	13
WPC-LR	↑ 8 ± 5	↓ 17 ± 11 *	15
WPC-MA	↓ 29 ± 7 ***	↓ 36 ± 7 ***	12
WPH-P	↑ 19 ± 8	↑ 24 ± 12	12
WPH-AT	↑ 65 ± 15 ***	↑ 131 ± 28 ***	12
AT-Active	↓ 11 ± 9	↓ 4 ± 12	10
AT-Inactive	↑ 6 ± 13	↓ 21 ± 11	9

Data show the mean percent change between 5 and 15 min of exposure relative to the pretreatment control, for tension (g) and frequency (contractions per minute) from ≥10 preparations (from ≥6 animals) and SEM. Asterisks indicate statistical significance of each treatment relative to pretreatment controls (* *p* < 0.05, *** *p* < 0.001). WPC-L, whey protein concentrate—lactic acid; WPC-LR, whey protein concentrate—lactic acid and rennet; WPC-MA, whey protein concentrate—mineral acid; WPH-P, whey protein hydrolysate—pancreatin; WPH-AT, whey protein hydrolysate—alcalase and thermoase. AT-Active, alcalase and thermoase active form; AT-Inactive, deactivated alcalase and thermoase.
